# Effect of Film Thickness and Defects on the Corrosion Behavior of Anodic Oxide Films on Aluminum Alloys

**DOI:** 10.3390/ma19030515

**Published:** 2026-01-28

**Authors:** Song Wang, Huwei Tao, Xianqin Zhuo, Linyue He, Pengfei Liu, Kai Dong, Bowei Zhang, Kui Xiao, Junsheng Wu

**Affiliations:** 1 Institute of Advanced Materials and Technology, University of Science and Technology Beijing, Beijing 100083, China; 2 Hainan International Commercial Aerospace Launch Co., Ltd., Wenchang 571300, China; 3 Xinjiang Joinworld Company Limited, Urumuqi 830000, China; 4 Shihezi Zhongjin Electrode Foil Company Limited, Shihezi 832000, China

**Keywords:** sulfuric acid-anodized film, aluminum alloy, medium-temperature sealing, high-temperature sealing, corrosion behavior

## Abstract

This study focuses on sulfuric acid-anodized films formed on 2A12 and 6061 aluminum alloys, in which the corrosion behavior of the oxide films under different film thicknesses, sealing methods, and defect states was investigated through neutral salt spray testing combined with surface morphology characterization and XRD analysis. The results indicate that the corrosion resistance of anodic oxide films is positively correlated with film thickness, while the anodized film on 2A12 aluminum alloy contains more cracks than that on 6061, which can readily serve as long-term corrosion initiation sites. Although the corrosion products of both alloys are identified as Al_2_O_3_ and AlO(OH), the oxide films on 6061 aluminum alloy exhibit higher compactness than those on 2A12 at all investigated thicknesses, resulting in superior resistance to neutral salt spray corrosion, and both sealing methods provide effective protection for the 6061 aluminum alloy substrate. This study provides experimental and theoretical references for the development and application of anodizing processes for aluminum alloys in chloride-containing marine environments.

## 1. Introduction

Aluminum alloys of the 2xxx and 6xxx series are widely applied in critical fields such as aerospace and marine vessels due to their excellent mechanical properties and good formability [1,2,3,4,5]; however, during service, they are highly susceptible to corrosion induced by environmental exposure, which not only causes material degradation but may also lead to performance failure and safety risks [6,7,8,9]. To extend the service life of aluminum alloys, surface treatment has become a key technological approach, among which anodizing provides an effective solution to address these challenges.

Anodizing is an electrochemical technique that produces dense, ordered, and porous Al_2_O_3_ films on aluminum alloy surfaces [10,11,12,13,14]. These films typically consist of an inner dense barrier layer and an outer porous layer; however, the porous layer facilitates the penetration of chloride ions, which can induce under-film corrosion [15,16,17,18]. Therefore, sealing treatments are commonly applied to improve corrosion resistance and prolong service life. The corrosion resistance of anodized films is strongly influenced by processing conditions, film thickness, and defect characteristics [19,20,21]. Zhao et al. [22] reported that anodized films on 2024 aluminum alloy develop cracks when exposed to elevated temperatures, and that the characteristics of these cracks, including their density, width, and depth, vary according to the substrate alloy used as well as the specific processing conditions under which the films are formed. Qian et al. [23] found that sealing treatments significantly enhance the corrosion resistance of anodized films in neutral salt spray tests. Liu et al. [24] observed that increasing current density and sulfuric acid concentration initially improves and then reduces film corrosion resistance, and that film thickness is positively correlated with corrosion resistance within a certain range. Delphine et al. [25] found that, when defects are present in anodized films on aluminum alloys, the electrochemical impedance of the films is significantly reduced.

Although extensive studies have been conducted on the fabrication processes, property regulation, and failure behaviors of anodized oxide films on aluminum alloy, most existing work has focused on the influence of a single factor on the corrosion resistance of the oxide layer. In chloride-containing corrosive environments, such as tropical marine conditions, the synergistic effects of film thickness and microstructural defects on the corrosion evolution of anodic oxide films remain poorly understood. Moreover, systematic investigations targeting aluminum alloys commonly used in marine engineering, such as 2A12 and 6061, are still relatively scarce. In chloride-containing corrosive environments such as tropical marine conditions, factors including the thickness, sealing state, and microstructural defects of anodized films on aluminum alloys have a significant impact on the corrosion resistance in marine environments. This study primarily investigates the effects of film thickness and microstructural defects on the corrosion behavior of anodized films on 2A12 and 6061 aluminum alloys. Anodized films of varying thicknesses were prepared using a sulfuric acid-anodizing process, and their surface microstructures were characterized by scanning electron microscopy (SEM) and laser confocal microscopy, among other techniques. The corrosion evolution of the films under neutral salt spray conditions was examined with respect to different thicknesses and defect states, while the corrosion morphologies and products after various exposure periods were analyzed to elucidate the mechanisms by which different types of defects promote localized corrosion. The study thereby clarifies the corrosion behavior of anodized films and provides both experimental evidence and theoretical guidance for the development and application of anodizing processes for aluminum alloys in chloride-containing marine environments.

## 2. Experimental Materials and Methods

### 2.1. Materials and Sample Preparation

2A12-T4 and 6061-T6 aluminum alloys were selected as the experimental materials, and their chemical compositions are listed in Table 1. Thin anodized films with thicknesses of 3–5 μm and thick anodized films with thicknesses of 15–20 μm were prepared on 2A12 and 6061 aluminum alloys. To investigate the effect of sealing treatments on the corrosion resistance of anodized films with different thicknesses, sulfuric acid-anodized samples of 2A12 and 6061 alloys were subjected to both medium-temperature and high-temperature sealing, with the specific process parameters summarized in Table 2. To suppress pH fluctuations caused by hydrolytic dissolution of the anodic oxide films at elevated temperatures, an acetic acid–sodium acetate buffer solution was used during the experiments.

### 2.2. Experimental Methods

The 2A12 and 6061 specimens with sulfuric acid-anodized films were placed in a JYWX-010 cyclic neutral salt spray chamber (Nanjing Huanke Experimental Equipment Co., Ltd., Nanjing, China) containing a 5 wt.% NaCl solution, and their corrosion morphologies were periodically examined after different exposure durations.

The rusted samples were immersed in an ultrasonic cleaning bath containing rust removal solution (HNO_3_, ρ = 1.42 g/mL) at room temperature for 5 min. Afterward, the samples were sequentially rinsed with deionized water and anhydrous ethanol. The depth of pitting corrosion was measured using a VK-X250 3D Laser Confocal Microscope (Keyence, Osaka, Japan) following the ASTM G46-94 (2005) standard [26], employing the microscope focal difference method. The maximum pitting depth of the samples was calculated based on the Gumbel distribution function model.

The surface and cross-sectional corrosion morphologies of the specimens were examined using a Regulus 8100 field emission scanning electron microscope (Bruker, Billerica, MA, USA) operated at 20 kV with a working distance of 15 mm. Backscattered electron mode was used to observe the cross-sectional morphology, while secondary electron mode was employed to analyze the surface morphology. The phase structure of the corrosion products was analyzed using a Dmax-RC rotating anode X-ray diffractometer (Rigaku, Akishima, Japan). A Cu Kα_1_ radiation source with a wavelength of 0.15418 nm was employed, and a sealed X-ray tube was used as the cathode. The operating conditions were set at 40 kV and 150 mA, with a scanning range of 2θ = 10–90°, a step size of 0.02°, and a scanning rate of 10°/min.

## 3. Results and Discussion

### 3.1. Microstructure of Anodized Films

**Figure 1:**
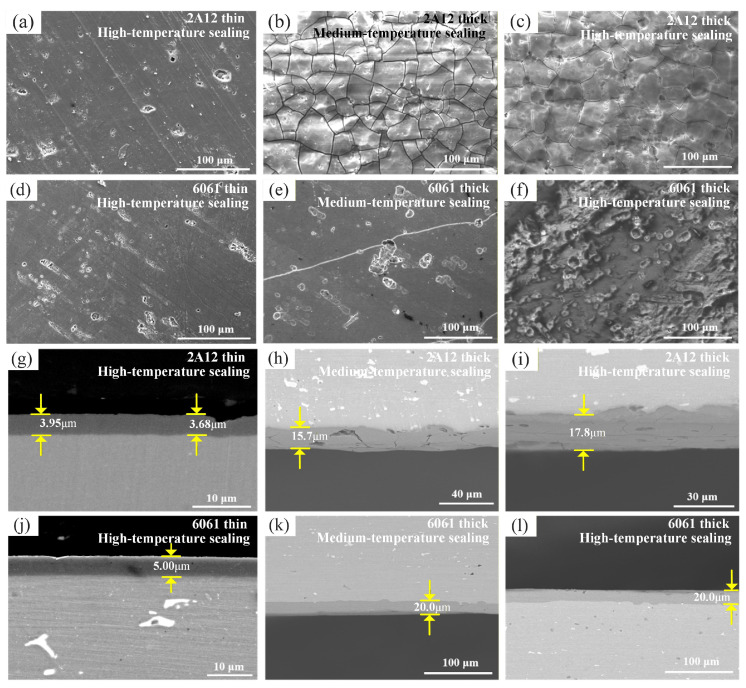
Surface and cross-sectional microstructure of anodized film samples (**a**,**g**) 2A12 thin anodized film; (**b**,**c**,**h**,**i**) 2A12 thick anodized film; (**d**,**j**) 6061 thin anodized film; (**e**,**f**,**k**,**l**) 6061 thick anodized film.

Figure 1 shows the surface and cross-sectional microstructure of six types of sulfuric acid-anodized film samples. From Figure 1a,d, it can be seen that the surface morphology of the 2A12 and 6061 thin anodized films treated by high-temperature sealing is similar, with the presence of pore defects but no significant cracks observed. After medium-temperature and high-temperature sealing, the surface of the 2A12 thick anodized film samples exhibits uniformly distributed defects and cracks, as shown in Figure 1b,c. The surface of the 6061 thick anodized film with medium-temperature sealing displays micron-sized pits or pores, along with a few cracks, while the high-temperature sealed sample shows more pore defects than the medium-temperature sealed group but no cracks, as shown in Figure 1e,f. From Figure 1g–l, it can be observed that the anodized film thicknesses of the high-temperature sealed 2A12 and 6061 thin anodized film samples are approximately 4 μm and 5 μm, respectively. The thicknesses of the medium-temperature and high-temperature sealed 2A12 thick anodized films are approximately 16 μm and 18 μm, respectively. The thickness of the medium-temperature and high-temperature sealed 6061 thick anodized films is approximately 20 μm.

### 3.2. Macroscopic Corrosion Morphology

**Figure 2:**
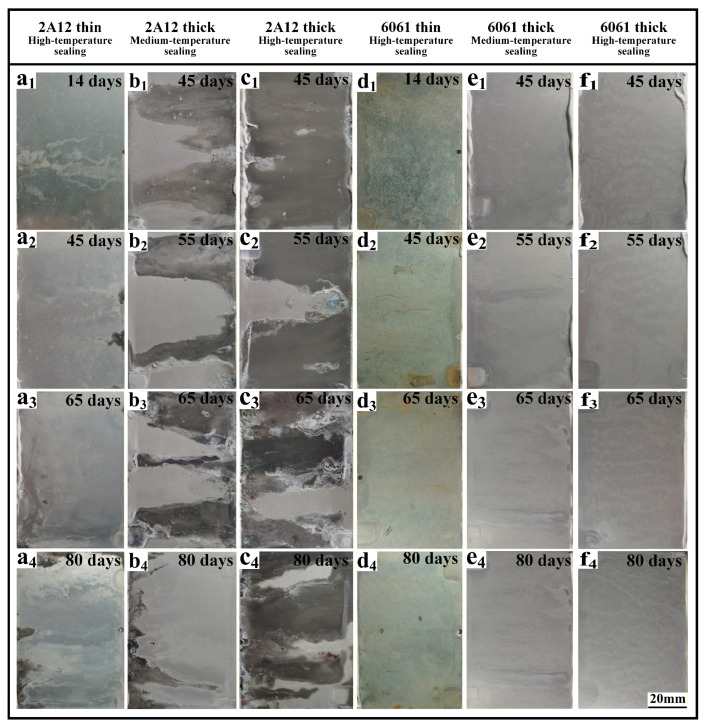
Macroscopic corrosion morphology of anodized film samples exposed to different durations of neutral salt spray environment (**a_1_**–**a_4_**) 2A12 thin anodized film; (**b_1_**–**b_4_**,**c_1_**–**c_4_**) 2A12 thick anodized film; (**d_1_**–**d_4_**) 6061 thin anodized film; (**e_1_**–**e_4_**,**f_1_**–**f_4_**) 6061 thick anodized film.

Figure 2 shows the macroscopic corrosion morphology of the 2A12 and 6061 anodized film samples after different durations of neutral salt spray testing. As seen in Figure 2(a_1_–a_4_), the pale yellow color on the surface of the 2A12 thin anodized film faded from localized areas as the exposure time increased. After 80 days, the overall color became noticeably lighter, and white flocculent corrosion products gradually covered the surface. Figure 2(b_1_–b_4_,c_1_–c_4_) show that, except for the edge diffusion areas, the surface of the 2A12 thick anodized films with medium-temperature and high-temperature sealing remained flat throughout the test, with no peeling of the oxide film or accumulation of corrosion products. As shown in Figure 2(d_1_–d_4_), the surface color of the 6061 thin anodized film lightened gradually with the increase in salt spray exposure time. After 65 days, scattered yellow-brown substances were observed in localized areas of the surface. By 80 days, these substances were distributed in a spotty pattern but did not form a continuous corrosion region. In contrast, the 6061 thick anodized film samples maintained a uniform surface color throughout the entire salt spray test, with no significant fading, corrosion products, or damage to the oxide film, thus preserving the initial appearance of the anodized layer, as shown in Figure 2(e_1_–e_4_,f_1_–f_4_). Overall, it can be concluded that the neutral salt spray corrosion resistance of the 6061 aluminum alloy anodized films was superior to that of the 2A12 alloy, with the 6061 thick anodized films demonstrating the most stable corrosion protection performance.

### 3.3. Microscopic Corrosion Morphology

**Figure 3:**
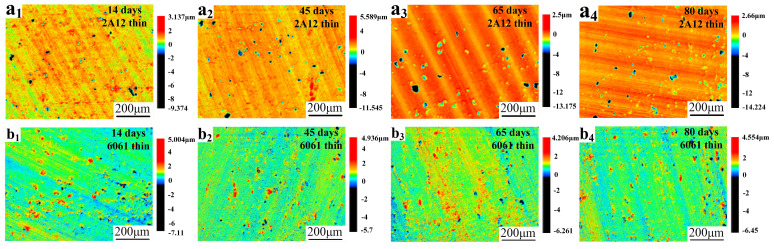
Pitting depth corrosion morphology of anodized film samples after corrosion product removal (**a_1_**–**a_4_**) 2A12 thin anodized film; (**b_1_**–**b_4_**) 6061 thin anodized film.

**Figure 4:**
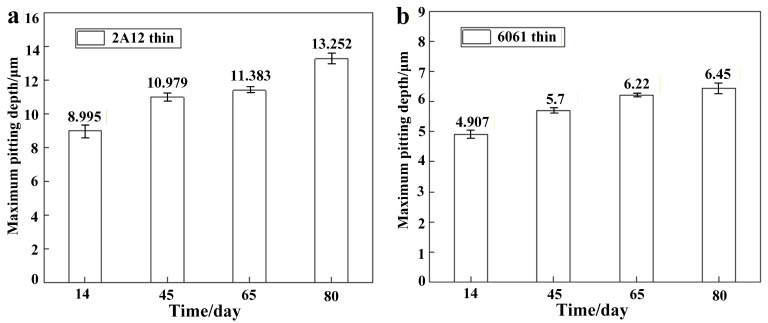
Maximum pitting depth statistics of samples under different exposure periods in a neutral salt spray environment (**a**) 2A12 thin anodized film; (**b**) 6061 thin anodized film.

Figure 3 shows the pitting depth morphology of 2A12 and 6061 thin sulfuric acid-anodized film samples after corrosion products were removed, as observed using a 3D Laser Confocal Microscope. Irregularly shaped pitting was observed on the surface of both samples, and with the increase in salt spray exposure time, the pit size showed a gradual expansion. Further analysis of the corresponding maximum pitting depth curves revealed that, as shown in Figure 4a, the pitting depth of the 2A12 thin anodized film sample continuously increased with exposure time. It rose from 8.995 μm after 14 days to 13.252 μm after 80 days, with the pits extending both longitudinally and laterally, and the corrosion area gradually coalescing. Figure 4b shows the maximum pitting depth curve for the 6061 thick anodized film sample under different exposure times in a neutral salt spray environment. The pitting depth also increased over time, from 4.907 μm at 14 days to 6.450 μm at 80 days. However, compared to the 2A12 sample, the extent and rate of pit expansion were significantly slower in the 6061 sample.

**Figure 5:**
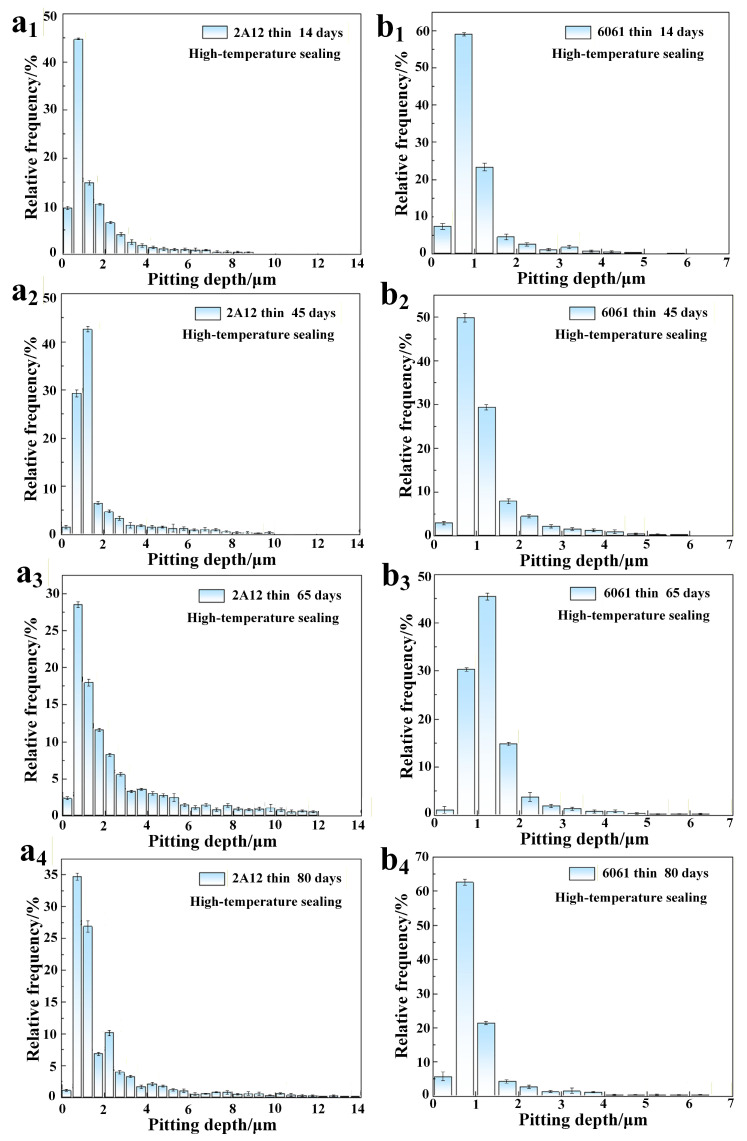
Pitting depth distribution of samples under different exposure periods in a neutral salt spray environment (**a_1_**–**a_4_**) 2A12 thin anodized film; (**b_1_**–**b_4_**) 6061 thin anodized film.

Figure 5 shows the pitting depth distribution of 2A12 and 6061 thin anodized film samples after exposure to different periods in a neutral salt spray environment. It can be observed that, as the corrosion time increases, the main distribution range of the pitting depth for the 2A12 thin anodized film sample remains in the 0–2.00 μm range. However, the proportion of pits in the 2.00–4.00 μm range shows a marked increase. After 80 days of corrosion, the proportion of pits deeper than 9.00 μm also increases, indicating that the longitudinal spread of pitting has expanded. In contrast, the pitting depth distribution for the 6061 thin anodized film sample shows a smaller overall change compared to the 2A12 sample. Only minor expansions in depth are observed within each exposure period, reflecting a more gradual longitudinal development of pitting.

**Figure 6:**
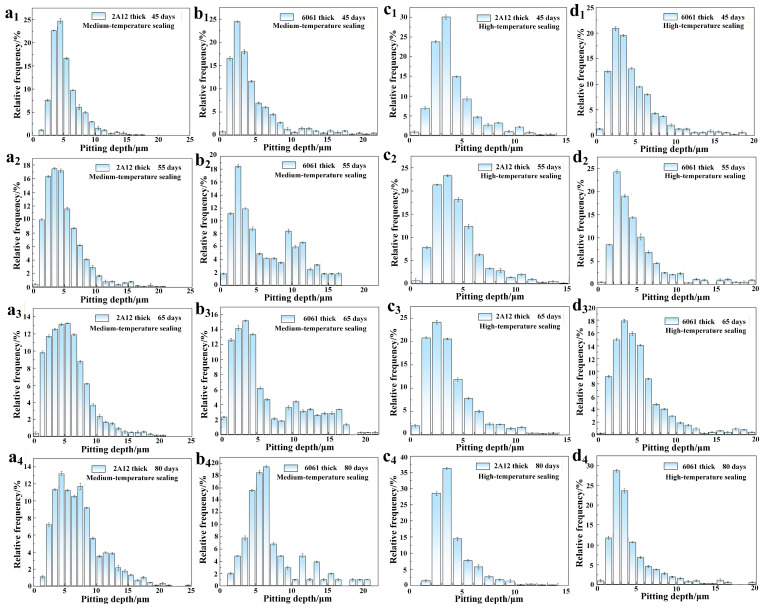
Pitting depth distribution of samples under different exposure periods in a neutral salt spray corrosion environment (**a_1_**–**a_4_**,**c_1_**–**c_4_**) 2A12 thick anodized film; (**b_1_**–**b_4_**,**d_1_**–**d_4_**) 6061 thick anodized film.

Figure 6 shows the pitting depth distribution of 2A12 and 6061 thick anodized film samples with medium-temperature and high-temperature sealing processes after exposure to different periods in a neutral salt spray environment. As shown in Figure 6(a_1_–a_4_,b_1_–b_4_), with the increase in corrosion time, the pitting depth of the 2A12 and 6061 thick anodized film samples with medium-temperature sealing gradually deepens, and the overall change is relatively small. As shown in Figure 6(c_1_–c_4_,d_1_–d_4_), similar trends are observed for the high-temperature sealed 2A12 and 6061 thick anodized film samples. The pitting depth slightly increases with longer exposure time, but the distribution changes are not significant. Combining the surface morphology after corrosion product removal with the pitting depth statistics, it can be concluded that, as the salt spray exposure time increases, the corrosion degree of the samples progressively intensifies, with both the lateral and longitudinal expansion of the pitting observed.

**Figure 7:**
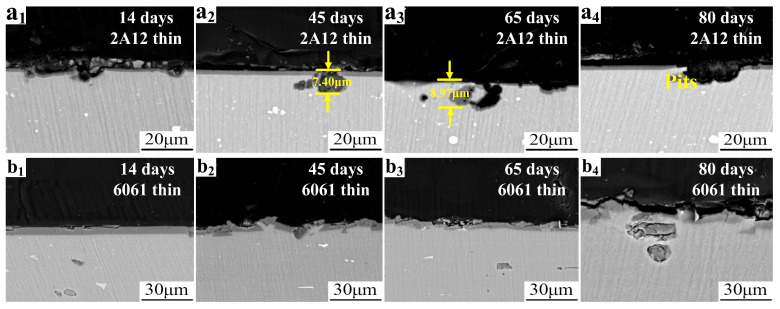
Cross-sectional microstructure of samples under different exposure periods in a neutral salt spray corrosion environment (**a_1_**–**a_4_**) 2A12 thin anodized film; (**b_1_**–**b_4_**) 6061 thin anodized film.

Figure 7 shows the cross-sectional microstructure of 2A12 and 6061 thin anodized film samples with high-temperature sealing after different periods of neutral salt spray exposure. As shown in Figure 7(a_1_–a_4_), for the 2A12 thin anodized film sample, after 14 days of corrosion, only small, shallow pits were observed on the surface. After 45 days of corrosion, the maximum pit depth reached 7.4 μm, accompanied by the aggregation of smaller pits. As cross-sectional observations only reflect local corrosion features along the cut plane, this value is slightly lower than the global maximum pit depth measured by 3D laser confocal microscopy. This discrepancy arises from the different measurement dimensions of the two techniques. As the corrosion time increased, the pits gradually penetrated through the entire anodized film and began to infiltrate the substrate. By 80 days, noticeable substrate pitting had formed. As shown in Figure 7(b_1_–b_4_), the corrosion progression for the 6061 thin anodized film sample was more gradual. After 14 days, only shallow depressions appeared on the surface of the anodized film. After 45 days, cracks and shallow pits appeared on the surface, and the integrity of the oxide film began to be compromised. However, by 80 days, only a few pits had penetrated through the oxide film to the substrate, significantly fewer than those observed in the 2A12 thin anodized film sample.

**Figure 8:**
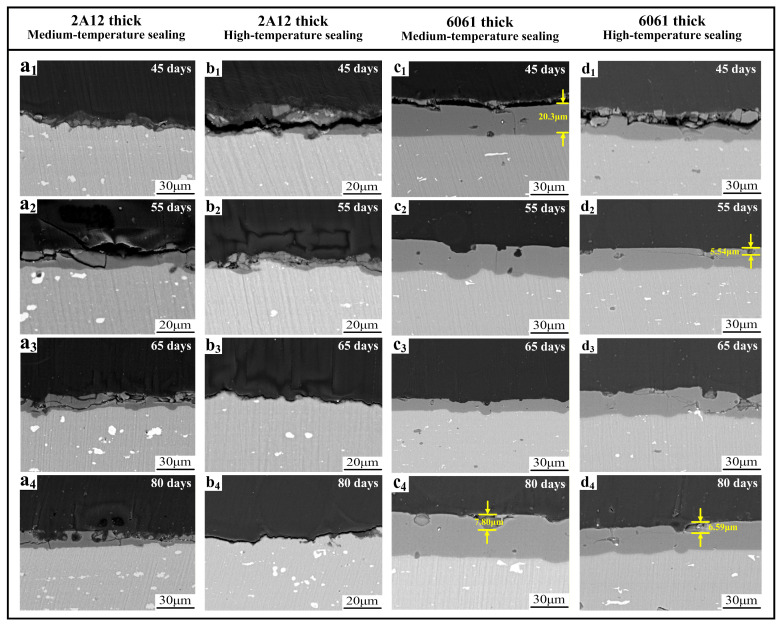
Cross-sectional microstructure of samples under different exposure periods in a neutral salt spray environment (**a_1_**–**a_4_**,**b_1_**–**b_4_**) 2A12 thick anodized film; (**c_1_**–**c_4_**,**d_1_**–**d_4_**) 6061 thick anodized film.

Figure 8 shows the cross-sectional microstructure of 2A12 and 6061 thick anodized film samples with medium-temperature and high-temperature sealing after different exposure periods in a neutral salt spray environment. As shown in Figure 8(a_1_–a_4_), for the 2A12 thick anodized film with medium-temperature sealing, the integrity of the anodized film was compromised in each exposure period. Shallow pitting was observed at the oxide–substrate interface, but the pits did not penetrate the film and reach the substrate. As shown in Figure 8(b_1_–b_4_), for the 2A12 thick anodized film with high-temperature sealing, the oxide film gradually thinned over time. The surface displayed small pits of varying depths, and the film exhibited numerous cracks. However, no pits extending from the cracks to the substrate were observed within the 80-day period. In medium-temperature sealing (nickel salt system), pore closure is achieved by the formation of fine nickel hydroxide precipitates within the film pores, which bind tightly to the alumina matrix and effectively block Cl^-^ penetration. In contrast, high-temperature sealing produces hydrated alumina through hydrolysis, which has a relatively high volumetric expansion and tends to generate internal stresses within the film. Under prolonged salt spray exposure, stress release can induce cracking and spalling, leading to film thinning [27,28]. In contrast, as shown in Figure 8(c_1_–c_4_,d_1_–d_4_), the 6061 thick anodized film samples with both medium-temperature and high-temperature sealing showed better oxide film integrity. Only shallow semicircular pits and a few cracks were observed, and no corrosion morphology penetrating the oxide film to the substrate was detected. Overall, the corrosion extent was less severe for the 6061 samples. In summary, the pitting behavior of the 2A12 and 6061 thin anodized film samples showed more pronounced changes. The 2A12 thick anodized film sample suffered from film damage in the neutral salt spray environment, while the 6061 thick anodized film sample exhibited milder pitting with better oxide film integrity, which is consistent with the 3D laser confocal characterization results.

After 80 days of exposure to a salt spray environment, significant differences in the corrosion behavior of the different 2A12 aluminum alloy samples were observed. The 2A12 thin anodized film sample exhibited localized corrosion that penetrated from the oxide film surface to the substrate. The pits expanded laterally and longitudinally, forming typical corrosion features that penetrated through the film. In contrast, the 2A12 thick anodized film samples with medium-temperature and high-temperature sealing did not show such penetrating pits. Although these samples exhibited numerous cracks in the oxide film, the film thickness was four to five times that of the 2A12 thin anodized film, effectively blocking the penetration of corrosive media and enhancing the protection of the substrate. In some periods of the 2A12 thick anodized film samples, the cross-sectional morphology revealed localized thinning or absence of the oxide film. This may be attributed to the presence of copper particles in the 2A12 alloy, which can reduce the adhesion between the oxide film and substrate during the early stages of anodizing. Additionally, this type of oxide film has a higher solubility in acidic salt spray environments, leading to localized film degradation. For the 6061 aluminum alloy samples, corrosion development was more gradual. The 6061 thin anodized film sample exhibited pitting as the salt spray exposure time increased, but both the number and depth of the pits were smaller than those observed for the same exposure periods in the 2A12 thin anodized film sample. The 6061 thick anodized film samples with medium-temperature and high-temperature sealing performed better, showing no obvious corrosion marks on the macroscopic morphology. On the microscopic level, only a few shallow pits were observed. The effect of the sealing methods on corrosion resistance was minimal.

Comparing the overall corrosion behavior of the two aluminum alloys, it is evident that the corrosion development trend of their anodized films is similar. However, the corrosion resistance of the 6061 aluminum alloy after sulfuric acid-anodizing is significantly superior to that of the 2A12 aluminum alloy. From the perspective of alloy composition, 6061 is an Al-Mg-Si alloy, which has fewer intermetallic particles or secondary phases, thereby reducing the potential corrosion initiation sites. From the standpoint of the oxide film characteristics, particularly for the 6061 thick anodized film samples, only a few pits or void defects were observed, with no cracks. Compared to the 2A12 thick anodized film samples processed under the same conditions, the 6061 samples exhibited superior uniformity and compactness, which is the key factor contributing to the enhanced corrosion resistance of anodized 6061 aluminum alloy.

### 3.4. Corrosion Product Analysis

**Figure 9:**
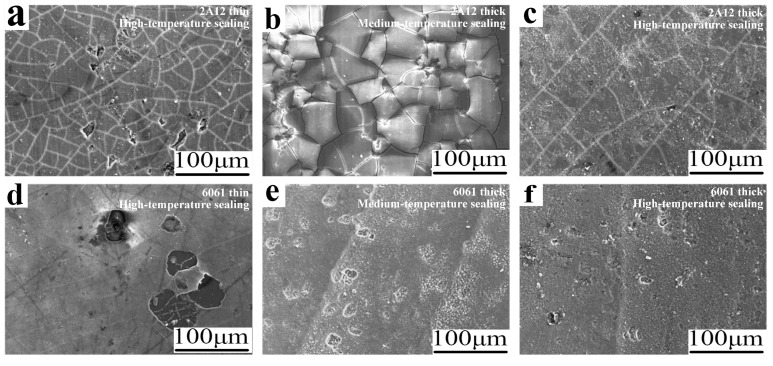
Surface microstructure of anodized film samples after 80 days of exposure to neutral salt spray environment (**a**) 2A12 thin anodized film; (**b**,**c**) 2A12 thick anodized film; (**d**) 6061 thin anodized film; (**e**,**f**) 6061 thick anodized film.

Figure 9 shows the SEM microstructure of the surface of six sulfuric acid-anodized film samples after 80 days of exposure to neutral salt spray corrosion. The surface of the 2A12 samples is uniformly covered with numerous prominent cracks and a small amount of irregular, particulate corrosion products. However, the crack characteristics on the surface of different 2A12 samples vary. The cracks in the medium-temperature and high-temperature sealed 2A12 samples differ in size, shape, and number, as shown in Figure 9a–c. As shown in Figure 9d, the surface of the 6061 thin anodized film sample exhibits faint microcracks, with localized damage to the anodized film due to corrosion. As shown in Figure 9e,f, the microstructures of the 6061 thick anodized film samples with medium-temperature and high-temperature sealing are highly similar. Only NaCl particles are enriched at the original pits and voids, creating a grayish overlay on the surface. Overall, the microscopic corrosion forms for all six samples are predominantly pitting corrosion, with no large-scale accumulation of corrosion products observed.

**Figure 10:**
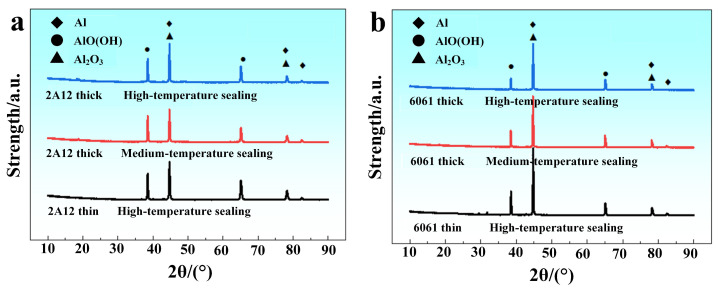
XRD spectra of corrosion products on anodized film samples after 80 days of neutral salt spray exposure (**a**) 2A12 sample; (**b**) 6061 sample.

Figure 10 presents the XRD spectra of the corrosion products on the surface of 2A12 and 6061 anodized film samples after 80 days of exposure to neutral salt spray corrosion. Figure 10a,b correspond to the 2A12 and 6061 sulfuric acid-anodized film samples, respectively. From the diffraction peak characteristics, it can be seen that the diffraction peaks of different samples within the same series are highly consistent, indicating that the corrosion product compositions of the 2A12 or 6061 samples are quite uniform, with Al_2_O_3_ and AlO(OH) being the predominant phases. Further comparison of the relative intensities of the diffraction peaks reveals that the AlO(OH) characteristic peak in the 2A12 thin anodized film with high-temperature sealing is slightly stronger than that in the 2A12 thick anodized film with medium-temperature and high-temperature sealing. For the 6061 thin anodized film sample, the relative intensity of the Al_2_O_3_ characteristic peak is significantly higher than that of the 6061 thick anodized film samples with medium-temperature and high-temperature sealing, reflecting slight differences in the corrosion product content between the samples.

In this study, the corrosion behaviors of six types of sulfuric acid-anodized films on 2A12 and 6061 alloys were systematically investigated, clarifying the relationship between the corrosion performance of the films and their microstructures. Based on these findings, corrosion protection using anodic oxide films should involve precise control of anodizing parameters to fabricate films dominated by highly dense and stable passivation phases, guided by XRD phase analysis; elimination of microstructural defects through organic–inorganic composite sealing or steam sealing techniques; and incorporation of corrosion-resistant ion modifications to enhance interfacial adhesion and resistance to localized corrosion. Future research could focus on revealing phase evolution and interfacial failure mechanisms of anodic films in corrosive media using in situ XRD combined with electrochemical impedance spectroscopy, conducting accelerated corrosion tests under multi-condition scenarios such as acidic, alkaline, saline, and wet–dry cycles to develop predictive models for film service life, and developing chromium-free, low-energy, environmentally friendly anodizing processes while exploring synergistic protection mechanisms with organic coatings and cathodic protection, thereby providing theoretical support and technical pathways for the industrial application of anodic oxide films in corrosion protection.

## 4. Conclusions

This study investigated the corrosion behavior of six sulfuric acid-anodized film samples of 2A12 and 6061 aluminum alloys through neutral salt spray tests, revealing the corrosion behavior changes in the oxide films under different film thicknesses and defect conditions. The corrosion morphology and corrosion products were analyzed over different exposure periods. The conclusions are as follows:

1. The corrosion resistance of both 2A12 and 6061 aluminum alloys increased with the thickness of the anodized film. The pit depth of 2A12 and 6061 thin anodized films (3–5 μm) significantly increased with the salt spray exposure period, whereas the pit development of the thick anodized films (15–20 μm) for both alloys was more gradual, and no corrosion penetrated through the film to the substrate.
2. The 2A12 thick anodized film (15–20 μm) exhibited cracks and void defects, with poor adhesion between the film and the substrate, making cracks more prone to long-term corrosion initiation. The 6061 thick anodized film (15–20 μm) contained only a small number of void defects and exhibited superior uniformity and compactness. The film integrity remained good throughout, providing more stable protection to the substrate.
3. The corrosion resistance of 6061 aluminum alloy is superior, and both sealing methods provided good protection to the 6061 alloy substrate. The corrosion products of both 2A12 and 6061 samples consisted of Al_2_O_3_ and AlO(OH), but the 6061 samples generated fewer corrosion products and exhibited overall better resistance to neutral salt spray corrosion.

## Figures and Tables

**Table 1:** Chemical composition of 2A12-T4 and 6061-T6 aluminum alloys (mass fraction/%).

Grade	Si	Fe	Cu	Mn	Mg	Ni	Cr	Zn	Ti	Al
2A12-T4	0.5	0.5	3.8~4.9	0.3~0.9	1.2~1.8	0.1	-	0.3	0.15	Bal.
6061-T6	0.4~0.8	0.7	0.15~0.4	0.15	0.8~1.2	-	0.04~0.35	0.25	0.15	Bal.

**Table 2:** Sulfuric acid-anodizing process conditions for 2A12 and 6061 samples.

Anodizing	Medium-Temperature Sealing	High-Temperature Sealing
H_2_SO_4_: 165–170 g/L Al^3+^: 10 g/L 18 V 19–21 °C 10–40 min	Ni^2+^: 1.0–1.1 g/L, NiSO_4_ pH = 5.45 60 °C 20 min	deionized water pH = 5.5–6.5 97–100 °C 60 min

## Data Availability

The original contributions presented in this study are included in the article. Further inquiries can be directed to the corresponding authors.
